# The Influence of Sociodemographic Characteristics and Prior Palliative Care Education and Experience on Undergraduate Nursing Students’ Attitudes Toward End-of-Life Care: A Cross-Sectional Study

**DOI:** 10.3390/healthcare14111489

**Published:** 2026-05-27

**Authors:** Essa Hakamy

**Affiliations:** Department of Nursing Administration and Education, College of Nursing, King Saud University, Riyadh P.O. Box 642, Saudi Arabia; ehakamy@ksu.edu.sa; Tel.: +966-118061627

**Keywords:** palliative care, knowledge, attitudes toward end-of-life care, nursing students, end-of-life care, PCQN, FATCOD-B

## Abstract

**Highlights:**

**What are the main findings?**

- Among 147 Saudi undergraduate nursing students, palliative care knowledge was associated with attitudes toward end-of-life care, with higher levels of knowledge being associated with more positive attitudes.
- Formal palliative care education was also associated with attitudes toward end-of-life care, whereas demographic and academic factors, such as age, sex, grade point average, and academic year were not consistently associated.

**What are the implications of the main findings?**

- These findings support strengthening and integrating structured palliative care education within undergraduate nursing curricula in Saudi Arabia.
- Formal education appears to be more influential than clinical exposure alone, suggesting a need for earlier, more consistent, and practice-oriented training in end-of-life care.

**Abstract:**

**Background:** The need for palliative care (PC) is increasing globally. As frontline healthcare workers, nurses play a pivotal role in providing PC to end-of-life (EOL) patients. Therefore, undergraduate nursing students must be adequately prepared to provide evidence-based, compassionate, and comprehensive care to patients during their terminal phase of life. Herein, we aimed to assess the knowledge and attitudes of Saudi undergraduate nursing students toward palliative and EOL care. **Methods:** This was a cross-sectional, correlational study conducted on 147 undergraduate nursing students from various regions of Saudi Arabia. The participants completed the Palliative Care Quiz for Nurses (PCQN) and Frommelt Attitudes Toward Care of the Dying-Form B scale (FATCOD-B) to assess their knowledge and attitudes toward EOL care, respectively. **Results:** Higher PCQN scores were significantly associated with higher FATCOD-B scores, suggesting that nursing students with higher levels of PC knowledge had more positive attitudes toward EOL care (r = 0.205, *p* = 0.013). Additionally, PC knowledge and formal PC education significantly predicted nursing students’ attitudes toward EOL care (β = 0.187, *p* < 0.05; β = −0.185, *p* < 0.05, respectively). The model explained 7.6% of the variance in nursing students’ attitudes toward EOL care (R^2^ = 0.076, *p* < 0.01). **Conclusions:** PC education remains inadequately integrated into undergraduate nursing curricula worldwide, despite nurses being at the forefront of EOL care. Embedding PC concepts early in nursing programs is crucial to build a workforce capable of meeting growing EOL care demands with genuine knowledge and confidence.

## 1. Introduction

The need for palliative care (PC) is increasing worldwide. As frontline healthcare workers, nurses play a pivotal role in providing PC to end-of-life (EOL) patients. It is estimated that 56.8 million people worldwide require PC annually; however, only 14% of these patients receive PC [1]. Although unmet PC needs are most commonly found among people living in low- and middle-income countries, there are concerns about gaps in PC education for undergraduate nursing students across all income levels. In Saudi Arabia, a country with a high-income economy, PC is still at a relatively early stage, having been introduced only about 20 years ago and not yet well established in the healthcare system [2]. However, EOL care is not only legally and religiously acceptable in Saudi Arabia but its ethics are grounded on the principles of the Holy Qur’an [2]. In Saudi Arabia, decisions regarding EOL care are made under Islamic law (Islamic jurisprudence), which provides specific terminology in the Qur’an to guide and resolve disputes about EOL care for terminally ill patients [3].

The World Health Organization defines PC as an approach to improve the quality of life of patients with life-threatening illnesses. It involves early identification, accurate assessment, and treatment to relieve and/or prevent suffering, pain, and other problems. It is integrated with curative treatment at any stage of a life-threatening illness. EOL care is a PC component that focuses specifically on care provision during a patient’s final phase of life [4]. Thus, it is provided to patients only when death is considered imminent, typically within weeks or months, and it refers to the provision of holistic support, dignity, and comfort during the patients’ terminal phase [5].

Nurses represent the largest proportion of frontline healthcare workers. They are instrumental in providing palliative and EOL care. Therefore, undergraduate nursing students should be adequately prepared to provide evidence-based, compassionate, and comprehensive care to patients during their terminal phase of life. Nevertheless, many nursing students report low confidence in delivering psychosocial and spiritual support, engaging in communication about death, and providing symptom management [6,7]. This may be related to a theory-focused and fragmented undergraduate curriculum on PC. The duration of undergraduate nursing programs in Saudi Arabia is usually 4 years, followed by 1 year of internship, and the programs are accredited by the Saudi Commission for Health Specialties [8]. Despite this structured pathway, PC is not well integrated in some nursing education programs, and many students are found to have no formal PC education upon graduation from nursing training programs [9].

Saudi Arabia acknowledges PC principles within its national nursing standards cited in the Saudi Palliative Care National Clinical Guidelines for Oncology [2]. These guidelines ensure that every patient with cancer in Saudi Arabia receives the best possible oncological care throughout the course of the disease. However, most nurses in Saudi Arabia do not fully understand the overarching concept of PC [10]. Although nursing students typically gain experience in general clinical care, their preparedness for palliative and EOL care specifically may vary, and this aspect warrants further investigation [10]. Thus, it is imperative to revise and strengthen undergraduate nursing curricula in Saudi Arabia, given the increasing burden of life-threatening illnesses. This is a prerequisite for delivering high-quality EOL care to patients in Saudi Arabia.

Several international studies have examined nursing students’ attitudes toward and knowledge of EOL and PC. However, there is a notable dearth of research assessing undergraduate nursing students’ knowledge of and attitudes toward palliative and EOL care. In addition, context-specific data on palliative and EOL care in Saudi Arabia are scarce, owing to service limitations and social norms [11]. Therefore, a comprehensive understanding of Saudi undergraduate nursing students’ knowledge, attitudes, and exposure to PC will inform targeted educational interventions, support evidence-based policy formulation, and guide nursing curriculum development in Saudi Arabia.

Additionally, Saudi Arabia faces the challenge of unprepared and uncoordinated clinical facilitators [12]. This stems from the diversity of the healthcare workforce. The workforce comprises individuals with a wide array of educational, linguistic, and cultural backgrounds [13]. This has left a critical gap in our understanding of undergraduate nurses’ preparedness at the pre-licensing level. Based on sociocultural diversity, higher education in Saudi Arabia requires a contextually grounded approach.

### 1.1. Purpose and Significance of the Study

In this study, we assessed the knowledge and attitudes of Saudi undergraduate nursing students toward palliative and EOL care. These elements are pivotal in identifying the necessary gaps in the nursing curriculum and for providing evidence-based recommendations regarding PC education. The significance of this study is that its findings can help enhance competency-based education and inform curriculum reforms in Saudi Arabia, thereby aiding in the development of a competent nursing workforce.

Therefore, the aims of this study were: (1) to describe the sociodemographic characteristics and factors associated with PC knowledge and attitudes toward EOL care among undergraduate nursing students; (2) to examine the relationship between selected sociodemographic characteristics and knowledge and attitudes toward palliative and EOL care among undergraduate nursing students; and (3) to examine the variance in the attitudes toward EOL care among undergraduate nursing students explained by selected sociodemographic characteristics and PC knowledge.

### 1.2. Theoretical Framework

This study is based on the theory of planned behavior (TPB) [14], which states that attitudes, subjective norms, and perceived behavioral control influence behavior. Based on the TPB, the purpose of this study was to identify the factors that affect these attitudes, in order to provide a basis for developing educational programs and curricula by focusing on these factors in Saudi nursing education.

## 2. Materials and Methods

### 2.1. Study Sample and Design

This cross-sectional correlational study was conducted to determine the factors associated with PC knowledge and attitudes toward EOL care among undergraduate nursing students. Data were collected using a convenience sampling approach. This study targeted undergraduate nursing students in the third and fourth year or internship stage of the Bachelor of Science in Nursing (BSN) program across different regions of Saudi Arabia. The inclusion criteria for the participants were as follows: (1) undergraduate nursing students in the third year, fourth year, and practicum (internship stage) who were enrolled in the BSN program at any school of nursing across the country, and (2) students with English literacy skills. The exclusion criteria were as follows: (1) students enrolled in the first and second years of the BSN program, (2) graduate nursing students, and (3) those who were not proficient in reading and writing English. In Saudi Arabian BSN programs, the practicum is a required, full-time supervised clinical training year completed after the four academic years, as required by the Saudi Commission for Health Specialties prior to professional licensure.

Using G*Power 3.1.9.6 software (Heinrich Heine University Düsseldorf, Düsseldorf, Germany), the minimum sample size required to achieve a statistical power ≥80% for an effect size of Cohen’s f^2^ = 0.15 was calculated to be 98 participants at a significance level of 0.05. After allowing for 10% missing data, the required sample size was calculated as 108 participants. A total of 147 participants were recruited for this study [15].

### 2.2. Data Collection

Institutional review board approval was obtained prior to data collection. Participants were recruited through an online invitation to complete surveys on Google Forms. The questionnaire link was shared with undergraduate nursing students through in-person announcements before the classes. To engage with a diverse range of participants from different regions, WhatsApp and email were used. In addition, the questionnaire was sent through organizational faculty email accounts to help recruit students who attended classes in person.

The initial page of the online questionnaire included a recruitment statement outlining the purpose of the study and the risks and benefits of participation. Additionally, statements regarding the maintenance of data privacy and confidentiality, as well as the participants’ right to withdraw from the study at any time, were included. Data were collected between February and March 2026.

### 2.3. Variables and Instruments

#### 2.3.1. Sociodemographic Characteristics

Data on sociodemographic characteristics, including age, sex, current academic year, current grade point average (GPA), whether the students had received any formal PC education, and whether they had previous experience in providing PC for patients or one of their family members, were collected from the online survey through the Google platform.

#### 2.3.2. Palliative Care Quiz for Nurses (PCQN)

The English version of the 20-item PCQN was used to assess students’ PC knowledge [16]. The overall PCQN score ranges from 0, indicating the “lowest level of knowledge” to 20, representing the “highest level of knowledge.” The final answers are coded as 1 = “correct,” 0 = “incorrect,” and “I do not know.” The reported Cronbach’s alpha coefficient of 0.67 indicates acceptable reliability for PCQN [17]. The Cronbach’s alpha coefficient in this study was 0.68, which meets the minimum threshold recommended for research instruments.

#### 2.3.3. Frommelt Attitudes Toward Care of the Dying Form-B Scale (FATCOD-B)

Third, the FATCOD-B was used to assess the students’ attitudes toward care of dying patients [18]. It consists of 9 items rated on a 5-point Likert scale, where 1 = “strongly disagree”, 2 = “disagree”, 3 = “uncertain”, 4 = “agree”, and 5 = “strongly agree.” The total score ranges from 9 to 45, with higher scores indicating more positive attitudes toward care of dying patients. All items refer to negative attitudes; therefore, they were revised to include positive content [19]. The mean scores were then calculated and converted to percentages, with higher percentages reflecting a more positive attitude toward care of dying patients. The reported Cronbach’s alpha coefficient for the FATCOD-B is 0.68. In this study, the scale demonstrated good internal consistency, with a Cronbach’s alpha coefficient of 0.87.

#### 2.3.4. Data Analysis

Descriptive statistics were used to summarize the study variables, with means and standard deviations reported for continuous measures (age, current GPA, and PCQN and FATCOD-B scores) and frequencies and percentages for categorical measures (sex, current academic year, formal PC education, and previous experience in providing PC for patients or a family member). Prior to analysis, the normality of variables was evaluated through examination of skewness and kurtosis values. All values fell within acceptable ranges (±2 for skewness and ±7 for kurtosis), supporting the use of parametric tests [20]. Pearson’s correlation coefficient was used to examine the strength and direction of the associations between continuous variables. Independent-sample *t*-tests and analysis of variance were conducted to assess group differences in GPA and the PCQN and FATCOD-B scores based on sex, academic year, formal PC education, and previous experience in providing PC for patients or a family member. Multiple linear regression analysis was performed to explore the predictive relationships among the sociodemographic and academic factors and the PCQN and FATCOD-B scores. In the analysis, a two-sided *p*-value < 0.05 was considered statistically significant. The assumptions of multiple linear regression (i.e., linearity, homoscedasticity, absence of autocorrelation, and absence of multicollinearity) were tested. The inspection of residual plots indicated that the assumptions of linearity and homoscedasticity were satisfied. The Durbin–Watson values ranged from 1.5 to 2.5, indicating no autocorrelation. There was no multicollinearity, with the variance inflation factor being within the normal range (<5). The residuals appeared to be normally distributed, as indicated by the Q-Q plot. All analyses were performed using SPSS v30 (IBM Corp., Armonk, NY, USA).

## 3. Results

Data from 147 students were included in the analysis. The mean age of the participants was 22.12 years (standard deviation [SD] = 1.22) (Table 1). Most participants were male (59.9%), and 40.1% were female. The mean GPA was 4.18 (SD = 0.46) on a 5-point scale. During the study period, 15% of the participants were in their third year, 47.6% were in their fourth year, and 37.4% were in the internship stage. Overall, 44.2% of the participants had received formal PC education, whereas 55.8% had not. In addition, 40.8% of the participants had previous experience in providing PC to patients or a family member, whereas 59.2% had no such previous experience. The mean PCQN and FATCOD-B scores were 6.68 (SD = 3.30) and 28.58 (SD = 8.36), respectively.

Table 2 presents the Pearson product-moment coefficients for the correlations among the study variables, including age, GPA, and the PCQN and FATCOD-B scores. The findings indicated that higher PCQN scores were significantly associated with higher FATCOD-B scores, assessing students’ attitudes toward EOL care, suggesting that nursing students with higher levels of PC knowledge demonstrated a more positive attitude toward EOL care (r = 0.205, *p* = 0.013). However, there were no associations of age with the students’ GPA or their PCQN or FATCOD-B scores.

Table 3 and Table 4 present the results of the independent sample *t*-test and analysis of variance among other study variables, including sex, academic year, formal PC education, and previous experience in providing PC to patients or a family member. The findings showed a significant association between the PCQN score and prior experience in providing PC to patients or a family member (*p* = 0.046). Furthermore, significant associations of the FATCOD-B score were found with formal PC education (*p* = 0.013) and previous experience in providing PC to patients or a family member (*p* = 0.030). However, there were no significant associations of sex and academic year with the PCQN or FATCOD-B scores.

Multiple linear regression analysis demonstrated that the overall model was statistically significant, F (2, 144) = 5.930, *p* = 0.003, accounting for 7.6% of the variance in the attitudes toward EOL care (Table 5). Both the PCQN score (B = 0.473, β = 0.187, 95% CI = 0.070–0.877) and formal PC education (B = 3.109, β = 0.185, 95% CI = 0.440–5.777) were significantly associated with nursing students’ attitudes toward EOL care (*p* < 0.05). These findings suggest that higher PCQN scores and formal PC education were associated with more positive attitudes toward EOL care among nursing students.

## 4. Discussion

Our findings revealed a significant relationship between nursing students’ PC knowledge, formal PC education, and EOL care provided to patients. Our analysis revealed that higher PCQN scores were significantly associated with more positive attitudes toward EOL care among nursing students. Students with higher PCQN scores demonstrated more positive attitudes toward EOL care, supporting the idea that knowledge shapes confidence in and readiness to care for dying patients. The findings were consistent with some studies that showed that an increase in knowledge was linked to a positive attitude toward caring for patients approaching death among Palestinian undergraduate nursing students. Similarly, another study showed a strong positive correlation between knowledge and attitude, measured by all items of the PCQN and FATCOD-B, respectively, which suggests that knowledge directly affects the attitudes of nursing students towards caring for dying patients [10,11,12,13,14,15,16,17,18,19].

The results obtained in this study are consistent with Ajzen’s TPB, which states that attitudes are formed based on knowledge and experiences [14]. The association between the students’ level of PC knowledge, their participation in PC training, and their attitudes toward EOL care—as reflected in the FATCOD-B score—is consistent with the TPB’s assertion that knowledge and experience lead to positive attitudes, which will eventually affect the students’ intention and actions in their future clinical practice. This result reinforces the significance of formal training in PC among undergraduate nursing students, as they must be equipped with adequate knowledge and experience to develop positive attitudes towards EOL care.

Formal PC education was another essential variable in this study. Participants who had received formal PC education demonstrated better scores for knowledge and attitude towards EOL care. This is supported by the findings of a study conducted in Najran, Saudi Arabia, which found that nursing students’ competence and attitudes are enhanced through structured PC education [10]. In contrast, reportedly, nursing students with no previous education on PC demonstrate poor knowledge scores, indicating that having exposure to a clinical setting is not enough to prepare students for EOL care [21]. These findings underscore the significance of incorporating PC training into undergraduate nursing curricula for improving knowledge and compassionate patient-oriented care.

Notably, previous experience in providing PC has been linked to better attitudes toward EOL care. However, this was not a significant predictor in the regression model in this study. This suggests that experience is insufficient on its own unless it is reinforced by organized education. Clinical exposure without guidance may lead to uncertainty or emotional discomfort, thereby limiting its positive impact. Previous studies support this finding, demonstrating that unstructured clinical experiences do not consistently improve competence unless paired with comprehensive education and reflection [22].

Our study did not find significant associations between demographic or academic variables, such as age, sex, GPA, and academic year, and PC knowledge or attitudes toward EOL care. In contrast, one study reported that views toward EOL care were significantly predicted by female sex and higher academic levels [23]. This discrepancy might be explained by the differences in the study samples. Our sample was relatively homogeneous; most participants were male, within a narrow age range, and had similar academic levels. Furthermore, the sample size may have made it more difficult to identify subtle changes between subgroups. Cultural and institutional differences may also play important roles. A study conducted demonstrated that attitudes toward death and dying are influenced by educational contexts and social norms [24].

The lack of association of PC knowledge or attitudes toward EOL care with GPA was also notable. This implies that general academic performance does not necessarily reflect PC competence. This finding supports the argument that PC requires specific training beyond the standard academic achievement. Emotional intelligence, communication skills, and ethical understanding are key components that are not always captured by GPA. However, research in this area has yielded inconsistent results. Some studies have found no correlation between academic performance (GPA) and PC competence [25]. However, other research found that students’ GPA was an independent factor associated with PC knowledge but not attitudes [19]. This discrepancy might be because of differences in the study samples; thus, more research is warranted.

### 4.1. Study Limitations

This study has some limitations. As this was a cross-sectional study, the ability to establish causality was limited. Although associations were identified between knowledge, education, and attitudes, it was not possible to determine cause-and-effect relationships. A longitudinal or experimental study would provide stronger evidence. Another possible limitation of this study is its reliance on self-reported measures, which may lead to response bias. Participants may have overestimated their knowledge, or they may have provided socially desirable responses [26]. This is especially true when dealing with sensitive topics, such as caring for dying patients, which could affect the accuracy of the findings.

More male students than female students participated in our study. This imbalance could limit the generalizability of the findings to settings with predominantly female nursing workforces. A previous study has reported sex differences in the attitudes towards death and EOL care [23]. Thus, the variability may not have been adequately represented in the current sample. In addition, the findings were based on convenience sampling, which could have been the reason for the observed unequal sex distribution, whereby male students were overrepresented compared with female students. This limits the generalizability of the results, as the sample may not be representative of the total Saudi undergraduate nursing student population. Moreover, variations in curriculum design, clinical exposure, and cultural values among institutions can profoundly affect students’ knowledge and attitudes. Thus, future research is recommended to compare different institutions’ outcomes regarding EOL care. Finally, the relatively small sample size may have lowered the statistical power to detect associations between some variables, such as age and academic level. Furthermore, the FATCOD-B has not been culturally validated in the Saudi context, and the results should be viewed with limited confidence until a culturally adapted instrument is created, as culturally relevant values and the role of Islam play an important factor in the attitudes of Saudi Arabian individuals towards death and dying.

Finally, it should be noted that the regression model explained only 7.6% of the variance in attitudes (R^2^ = 0.076), indicating a small effect size. The unexplained variance indicates that affective, spiritual, cultural, and experiential variables might impact attitudes towards EOL care among nursing students.

### 4.2. Implications for Future Research, Nursing Education, and Policy

The study findings have important implications for nursing education and policy. The findings emphasize the need to structure and incorporate PC education into undergraduate nursing curricula. This should include theoretical content and practical training to improve knowledge and build positive attitudes. Simulations, case-based learning, and supervised clinical experiences can help nursing students develop confidence in providing PC [27].

The results support the need for curriculum reform by policymakers and academic leaders, such as nursing college leaders. Nursing programs should align with national and international standards to guarantee that graduates are competent in PC. This will contribute to improving nursing students’ PC knowledge in order to meet the need to provide high-quality EOL care for patients. As countries and the world continue to accept and recognize the value of PC, modifying nursing curricula to meet these expectations is vital now more than ever. Standardized guidelines and competency frameworks can promote consistency among institutions.

Future studies should evaluate the effectiveness of educational interventions in enhancing nursing students’ PC knowledge and attitudes toward EOL care, particularly those related to psychological and emotional learning experiences, including coping strategies, emotional support, and positive experiences about EOL care and settings [28,29]. These efforts can improve clinical competence and resilience of students and future nurses and promote self-care. Qualitative studies can further explore students’ experiences and perceptions to provide deeper insights into the obstacles and enablers of learning in this area.

## 5. Conclusions

PC is an essential component of holistic patient-centered care; however, its integration into undergraduate nursing curricula remains inadequate worldwide. This study provides evidence that nursing students’ perspectives on EOL care are significantly influenced by formal PC education and PC knowledge. Students with higher knowledge levels and formal training in PC exhibited more positive attitudes toward EOL care. However, knowledge gaps remain, and not all variables are associated with attitudes toward EOL care. PC involves a comprehensive assessment of physical, psychosocial, and spiritual needs to anticipate and relieve suffering. Competencies must be developed early in undergraduate education rather than relying on clinical experience alone. Nurses are at the forefront of EOL care delivery, making their education critically important. Therefore, embedding PC concepts into undergraduate nursing programs is a crucial step toward building workforce with stronger knowledge and more positive attitudes toward EOL care.

## Figures and Tables

**Table 1:** Participant characteristics (N = 147).

Variable	Mean ± SD or n (%)
Age (y)	22.12 ± 1.22
Sex	
Male	88 (59.9%)
Female	59 (40.1%)
GPA ^a^	4.18 ± 0.46
Missing	0 (0%)
Academic year	
Third year	22 (15%)
Fourth year	70 (47.6%)
Internship	55 (37.4%)
Formal PC education	
Yes	65 (44.2%)
No	82 (55.8%)
Previous experience in providing PC for patients or a family member	
Yes	60 (40.8%)
No	87 (59.2%)
PCQN score	6.68 ± 3.30
FATCOD-B score	28.58 ± 8.36

^a^ Five-point scale. SD, standard deviation; GPA, grade point average; PC, palliative care; PCQN, Palliative Care Quiz for Nurses; FATCOD-B, Frommelt Attitudes Toward Care of the Dying Form-B scale.

**Table 2:** Pearson correlation matrix for the study variables.

Variables (r)	1	2	3	4
1. Age (y)	-			
2. GPA ^a^	−0.050	-		
3. PCQN score	−0.004	−0.025	-	
4. FATCOD-B score	−0.132	−0.078	0.205 *	-

^a^ Five-point scale. * *p* ≤ 0.05, GPA, grade point average; PCQN, Palliative Care Quiz for Nurses; FATCOD-B, Frommelt Attitudes Toward Care of the Dying Form-B scale.

**Table 3:** Independent-samples *t*-test results (mean differences in the PCQN and FATCOD-B scores by sex and formal PC education and previous experience in providing PC to patients or a family member).

	Variable	Mean ± SD	*t*	df	*p*	95% CI	
						LL	UL
PCQN score	Sex		0.842	146	0.401	−0.45	1.12
	Male	6.54 ± 3.11					
	Female	6.88 ± 3.57					
	Formal PC education		1.215	146	0.226	−0.28	1.60
	Yes	7.05 ± 3.07					
	No	6.39 ± 3.45					
	Previous experience in providing PC to patients or a family member		2.015	146	0.046 *	0.02	2.18
	Yes	7.33 ± 3.47					
	No	6.23 ± 3.11					
FATCOD-B score	Sex		1.087	146	0.279	−1.15	5.33
	Male	29.42 ± 7.83					
	Female	27.33 ± 9.01					
	Formal PC education		2.507	146	0.013 *	0.74	6.12
	Yes	30.5 ± 8.28					
	No	27.07 ± 8.15					
	Previous experience in providing PC to patients or a family member		2.198	146	0.030 *	0.27	4.93
	Yes	30.1 ± 9.22					
	No	27.50 ± 7.57					

* *p* ≤ 0.05. SD, standard deviation; df, degrees of freedom; CI, confidence interval; LL, lower limit; UL, upper limit; PC, palliative care; PCQN, Palliative Care Quiz for Nurses; FATCOD-B, Frommelt Attitudes Toward Care of the Dying Form-B scale.

**Table 4:** One-way analysis of variance results (mean differences in the PCQN and FATCOD-B scores by academic year).

	Variable	Mean ± SD	F Value	df	*p*	95% CI	
						LL	UL
PCQN score	Academic year		1.121	146	0.329	6.14	7.22
	Third year	7.18 ± 3.77					
	Fourth year	6.25 ± 3.16					
	Internship	7.01 ± 3.25					
FATCOD-B score	Academic Year		0.305	146	0.737	27.22	29.95
	Third year	27.59 ± 9.12					
	Fourth year	28.42 ± 8.03					
	Internship	29.18 ± 8.55					

SD, standard deviation; df, degrees of freedom; CI, confidence interval; LL, lower limit; UL, upper limit; PC, palliative care; PCQN, Palliative Care Quiz for Nurses; FATCOD-B, Frommelt Attitudes Toward Care of the Dying Form-B scale.

**Table 5:** Regression analysis of factors associated with attitudes toward end-of-life care (assessed using FATCOD-B).

Independent	B ^a^	Β ^b^	t	*p*	95% CI	
					LL	UL
PCQN score	0.473	0.187	2.320	0.022 *	0.070	0.877
Formal PC education	3.109	0.185	2.303	0.023 *	0.440	5.777
Model Summary	F (2, 144) = 5.930, R^2^ = 0.076, *p* = 0.003					

^a^, unstandardized coefficient; ^b^, standardized coefficient beta; * *p* < 0.05, CI, confidence interval; LL, lower limit; UL, upper limit; PCQN, Palliative Care Quiz for Nurses; FATCOD-B, Frommelt Attitudes Toward Care of the Dying Form-B scale.

## Data Availability

The raw data supporting the conclusions of this article will be made available by the authors on request.

## Abbreviations

The following abbreviations are used in this manuscript:

BSN	Bachelor of science in nursing
CI	Confidence interval
EOL	End-of-life
GPA	Grade point average
PC	Palliative care
PCQN	Palliative Care Quiz for Nurses
FATCOD-B	Frommelt Attitudes Toward Care of the Dying Form-B scale
TPB	Theory of planned behavior

## References

[1] Vélez-López A., Carmona-Torres J.M., López-González Á., Laredo-Aguilera J.A., Callado-Pérez D., Rabanales-Sotos J. (2024). Community-based interventions in people with palliative care needs: An integrative review of studies from 2017 to 2022. Healthcare.

[2] Abduldaiem A., Fouad Alem A., Saleh H., Al-Suhail A. (2023). Comparing no-show rates between palliative care and non-palliative care outpatient clinics at King Fahad Medical City, Riyadh, and KSA: A focus on oncology. Oncol. Radiother..

[3] Babgi A. (2009). Legal issues in end-of-life care: Perspectives from Saudi Arabia and United States. Am. J. Hosp. Palliat. Med..

[4] World Health Organization (2020). Palliative Care. https://www.who.int/news-room/fact-sheets/detail/palliative-care.

[5] National Institute on Aging (2021). What Are Palliative Care and Hospice Care? Bethesda (MD): National Institute on Aging. https://www.nia.nih.gov/health/hospice-and-palliative-care/what-are-palliative-care-and-hospice-care.

[6] Stephenson P., Hansen D., Lalani N., Biggs J. (2023). Nursing and medical students’ responses about end-of-life communication reveal educational opportunities for spiritual care. J. Nurs. Educ..

[7] Cheon J., You S.Y. (2022). Nursing students’ witnessed experience of patient death during clinical practice: A qualitative study using focus groups. Nurse Educ. Today.

[8] Aljohani K.A.S. (2020). Nursing education in Saudi Arabia: History and development. Cureus.

[9] Khraisat O.M., Hamdan M., Ghazzawwi M. (2017). Palliative care issues and challenges in Saudi Arabia: Knowledge assessment among nursing students. J. Palliat. Care.

[10] Elrefaey S.R.I., Osman A.M.A., Aljanabi H.M., Alzahrani M.J., Alhuraysi O.M.S., Idris A.M., Alhemairy M.A., Almutairi H.T., Taha W.H., Nageeb S.M. (2024). Enhancing Palliative Care Expertise among Nursing Students in Najran, Saudi Arabia: An Educational Intervention Study. Adv. Biol. Res..

[11] Alodhialah A.M., Almutairi M. (2025). Exploring palliative care access among older adults in Saudi Arabia: Implications for nursing practice. BMC Nurs..

[12] Almutairi A.F., McCarthy A., Gardner G.E. (2015). Clinical nursing teaching in Saudi Arabia: Challenges and suggested solutions. J. Nurs. Care.

[13] Rawas H.O., de Beer J., Abu Bakar S.A., Almutairi S., Jaafari N., Hazzazi H., Alzahrani A., Alghumuy R., Hadadi N., Alfahimi S. (2025). Barriers and facilitators to patient education among nurses in multicultural hospital settings: A cross-sectional study. Nurs. Rep..

[14] Ajzen I. (1991). The theory of planned behavior. Organ. Behav. Hum. Decis. Process..

[15] Faul F., Erdfelder E., Buchner A., Lang A.-G. (2009). Statistical power analyses using G*Power 3.1: Tests for correlation and regression analyses. Behav. Res. Methods.

[16] Ross M.M., McDonald B., McGuinness J. (1996). The palliative care quiz for nursing (PCQN): The development of an instrument to measure nurses’ knowledge of palliative care. J. Adv. Nurs..

[17] Chover-Sierra E., Martínez-Sabater A., Lapeña-Moñux Y.R. (2017). An instrument to measure nurses’ knowledge in palliative care: Validation of the Spanish version of Palliative Care Quiz for Nurses. PLoS ONE.

[18] Molinengo G., Loera B., Miniotti M., Leombruni P. (2021). Shortening the Frommelt attitude toward the care of the dying scale (FATCOD-B): A brief 9-item version for medical education and practice. J. Cancer Educ..

[19] Alwawi A.A., Abu-Odah H., Bayuo J. (2022). Palliative care knowledge and attitudes towards end-of-life care among undergraduate nursing students at Al Quds University: Implications for Palestinian education. Int. J. Environ. Res. Public Health.

[20] Kim H.Y. (2013). Statistical notes for clinical researchers: Assessing normal distribution (2) using skewness and kurtosis. Restor. Dent. Endod..

[21] Jothishanmugam A. (2025). Knowledge of undergraduate nursing students about palliative care. J. Educ. Health Promot..

[22] Alhaddar M., Falna H., Sultan H., Abu-Odah H., Khleif M., Najjar S. (2025). Towards enhancing palliative care competencies through comprehensive training for nurses and physicians in resource-limited settings: A cross-sectional study. BMC Nurs..

[23] Zahran Z., Hamdan K.M., Hamdan-Mansour A.M., Allari R.S., Alzayyat A.A., Shaheen A.M. (2021). Nursing students’ attitudes towards death and caring for dying patients. Nurs. Open.

[24] Qtait M., Alqaissi N. (2025). Attitudes of Palestinian nursing students toward family involvement in end-of-life care. Int. J. Palliat. Nurs..

[25] Prachyakoon N., Hounnaklang N., Win N., Tantirattanakulchai P. (2024). Exploring the factors related to knowledge of palliative care for patients with terminal cancer among nursing students: A cross-sectional study. Int. J. Nurs. Educ..

[26] Koo M., Yang S.-W. (2025). Questionnaire use and development in health research. Encyclopedia.

[27] Esteban-Burgos A.A., Moya-Carramolino J., Vinuesa-Box M., Puente-Fernández D., García-Caro M.P., Montoya-Juárez R., López-Morales M. (2024). Clinical simulation in palliative care for undergraduate nursing students: A randomized clinical trial and complementary qualitative study. Healthcare.

[28] Yoong S.Q., Wang W., Seah A.C.W., Kumar N., Gan J.O.N., Schmidt L.T., Lin Y., Zhang H. (2023). Nursing students’ experiences with patient death and palliative and end-of-life care: A systematic review and meta-synthesis. Nurse Educ. Pract..

[29] Dahò M. (2021). An exploration of the emotive experiences and the representations of female care providers working in a perinatal hospice: A pilot qualitative study. Clin. Neuropsychiatry.

